# Smoking during pregnancy and risk of abnormal glucose tolerance: a prospective cohort study

**DOI:** 10.1186/1471-2393-10-55

**Published:** 2010-09-17

**Authors:** Amy E Haskins, Elizabeth R Bertone-Johnson, Penelope Pekow, Elena Carbone, Renée T Fortner, Lisa Chasan-Taber

**Affiliations:** 1 Center for Outcomes Research and Evaluation, Maine Medical Center, Portland, ME, USA; 2 Division of Biostatistics & Epidemiology, Department of Public Health, School of Public Health & Health Sciences, University of Massachusetts, Amherst, MA, USA; 3 Department of Nutrition, School of Public Health & Health Sciences, University of Massachusetts, Amherst, MA, USA

## Abstract

**Background:**

Disturbances in glucose metabolism during pregnancy are associated with negative sequalae for both mother and infant. The association between smoking and abnormal glucose tolerance (AGT) remains controversial. Therefore, the aim of this study was to examine the relationship between smoking prior to and during pregnancy and risk of AGT.

**Methods:**

We utilized data from a prospective cohort of 1,006 Hispanic (predominantly Puerto Rican) prenatal care patients in Western Massachusetts. Women reported pre- and early pregnancy smoking at recruitment (mean = 15 weeks) and mid pregnancy smoking at a second interview (mean = 28 weeks). AGT was defined as > 135 mg/dL on the routine 1-hour glucose tolerance test (1-hr OGTT). We used multivariable regression to assess the effect of pre, early, and mid-pregnancy smoking on risk of AGT and screening plasma glucose value from the 1-hr OGTT.

**Results:**

In age-adjusted models, women who smoked > 0-9 cigarettes/day in pre-pregnancy had an increased risk of AGT (OR = 1.90; 95% CI 1.02-3.55) compared to non-smokers; this was attenuated in multivariable models. Smoking in early (OR = 0.48; 95% CI 0.21-1.10) and mid pregnancy (OR = 0.38; 95% CI 0.13-1.11) were not associated with AGT in multivariable models. Smoking during early and mid pregnancy were independently associated with lower glucose screening values, while smoking in pre-pregnancy was not.

**Conclusions:**

In this prospective cohort of Hispanic women, we did not observe an association between smoking prior to or during pregnancy and risk of AGT. Findings from this study, although based on small numbers of cases, extend prior research to the Hispanic population.

## Background

An estimated four to twelve percent of pregnancies are complicated by some degree of abnormal glucose intolerance (AGT), including gestational diabetes mellitus (GDM) as well as milder degrees of glucose tolerance [1]; rates are higher in Hispanic women as compared to non-Hispanic white women. Recently, the 7-year prospective Hyperglycemia and Adverse Pregnancy Outcome study found a consistent, linear increase in risk of adverse perinatal outcomes (cesarean section, fetal size, neonatal hypoglycemia, and fetal hyperinsulinemia) over the entire range of maternal blood glucose levels [2], not just with GDM, suggesting that identifying risk factors for milder degrees of disturbances in glucose metabolism is critical for maternal and offspring health.

Cigarette smoking remains common during pregnancy [3,4], but research examining the role of smoking as a risk factor for AGT is limited. Smoking has been associated with increased insulin resistance and type 2 diabetes in non-pregnant women [5]. However, whether smoking is a risk factor for glucose disturbances during pregnancy is less clear. Physiologic studies suggest that smoking may increase circulating levels of catecholamines, compounds involved in insulin resistance, and that nicotine may have direct toxic effects on pancreatic beta cell function [6-8].

Epidemiologic evidence supporting the role of smoking as a risk factor for GDM is limited and there is major heterogeneity in research design [9]. With few exceptions, the majority of studies have examined smoking as a dichotomous variable [10-15] and only five studies presented adjusted odds ratios [16-20]. Furthermore, only one prior study has been conducted in a Hispanic population [21]. There is some evidence that nicotine metabolism varies by race, leading to higher and lower nicotine exposure per cigarette among African-Americans and Chinese-Americans, respectively, although Hispanics appear to metabolize nicotine at the same rate as whites [22,23]. In addition, smoking patterns vary markedly among US Hispanic women according to country of origin, with Puerto Rican women having the highest rates of smoking during pregnancy compared to Hispanics from other countries [4]. One study found that 41% of female Puerto Rican smokers preferred menthol brands of cigarettes, and that menthol smokers may have larger inhaled volumes and higher cotinine levels than non-menthol smokers [24]. These characteristics of cigarette use among Hispanics may impact the effect of smoking on AGT in this population.

This is important as Hispanics are one of the fastest-growing segments of the US population, comprising 14.7% of the female population in 2008 [25]. Over 24% of all US births in 2007 were to Hispanics [26], representing the highest fertility rate of any race or ethnic group. Health status varies widely between Hispanic subgroups, with rates of adverse birth outcomes highest for Puerto Rican women [27].

Therefore, we prospectively examined the association between smoking during pre, early, and mid pregnancy and risk of AGT among a population of predominantly Puerto Rican prenatal care patients at a large tertiary care facility. We hypothesized that smokers during pre, early, or mid pregnancy would have an increased risk of AGT as compared to non-smokers. The current study contributes to the existing literature by examining the association between smoking during pregnancy, including cigarettes per day, and risk of AGT in a high-risk population of Hispanic prenatal care patients. To our knowledge, this is first prospective study of smoking and AGT in a Puerto Rican population.

## Methods

### Study Design

The Latina GDM Study was designed to examine physical activity and risk of GDM among Hispanic (predominantly Puerto Rican) women. Details of the study have been provided elsewhere [28]. Briefly, the study was based at the public clinic and midwifery practice of Baystate Medical Center, a large tertiary care facility in Western Massachusetts. Between September 2000 and December 2003, prenatal care patients who self-identified as Hispanic were recruited prior to the 24^th ^week of gestation (mean = 15 weeks of gestation, standard deviation (SD) = 5 weeks). Reasons for exclusions included age over 40 or under 16 years; non-Hispanic ethnic group; history of type 2 diabetes; hypertension or heart disease; chronic renal disease; non-singleton pregnancy; > 24 weeks of gestation at first interview; or prior participation in the study. All participants signed a written informed consent. The study was approved by the Institutional Review Boards of the University of Massachusetts-Amherst and Baystate Medical Center.

Bilingual interviewers collected information on current smoking status as well as smoking in the year prior to pregnancy. Information on sociodemographic characteristics and lifestyle factors was also collected. A second interview conducted in mid pregnancy (mean = 28 weeks, SD = 5 weeks) updated information on cigarette smoking. A subsample of participants completed a food frequency questionnaire (FFQ) at their mid-pregnancy visit. Medical and obstetrical history as well as clinical characteristics of the current pregnancy were abstracted from medical records after delivery.

A total of 1,231 prenatal care patients were enrolled in the Latina GDM Study. For the current analysis, we excluded women with a spontaneous or therapeutic abortion or preterm birth before 28 weeks (n = 48), women who did not deliver at Baystate (n = 123), and women not screened for GDM (n = 54), resulting in a sample size of 1,006 participants.

### Assessment of Cigarette Smoking

Cigarette smoking was assessed using questions designed by the Pregnancy Risk Assessment Monitoring System (PRAMS), a surveillance project of the Centers for Disease Control and Prevention [29]. Specifically, for the pre-pregnancy period, participants were asked how many cigarettes or packs of cigarettes were smoked on an average day (one pack = 20 cigarettes) in the year prior to pregnancy. Women were then classified as non-smokers (referent category) or smokers (> 0-9, or 10+ cigarettes per day) during the pre-pregnancy period. Participants also self-reported the number of cigarettes or packs smoked on an average day since pregnancy awareness ("early pregnancy" smoking). At the second interview, smoking since the last interview was collected ("mid pregnancy" smoking). For early pregnancy, women were classified as 1) non-smokers (i.e., non-smokers in both pre and early pregnancy), 2) former smokers (i.e., smokers in pre-pregnancy but non-smokers in early pregnancy), or 3) smokers in early pregnancy. For mid pregnancy, women were classified as 1) non-smokers (i.e., non-smokers in pre, early, and mid pregnancy), 2) former smokers (i.e., smokers in pre or early pregnancy but non-smokers in mid pregnancy), or 3) smokers in mid pregnancy. Women who reported being non-smokers in either the early or mid pregnancy periods but were missing smoking information for the previous time period(s) (n = 24 and n = 72, respectively), were categorized with former smokers for that previous period in order to preserve a reference group of confirmed non-smokers who did not smoke in any prior pregnancy period. Analyses were limited to the women with complete information on smoking during the pre (n = 912), early (n = 847), and mid (n = 697) pregnancy time periods, respectively. However, as a secondary analysis we restricted the analysis to participants with complete data in all three time periods.

### Assessment of Abnormal Glucose Tolerance

Prenatal care patients were screened for GDM between the 24^th ^and 28^th ^week of gestation as part of routine hospital protocol. The screening test consisted of administering a random (non-fasting) 50-g glucose load and a plasma glucose determination one hour later (1-hr OGTT). If the plasma glucose value was > 135 mg/dL, the woman was classified as having AGT in accordance with American Diabetes Association criteria [30]. We also evaluated the 1-hr OGTT plasma glucose values as a continuous outcome measure.

### Covariate Assessment

We collected information about potential risk factors for AGT. Maternal age, education level, and annual household income were assessed at the baseline interview. At this time interviewers also assessed language preference, birth place, and pregnancy behaviors including alcohol consumption and physical activity. Physical activity was assessed via the Kaiser Physical Activity Survey (KPAS) [31]. The KPAS provides a quantifiable index value ranging from 1 to 5 for each domain specific response for four domains of activity: household/caregiving, occupational, active living, and sports/exercise using the scoring procedures outlined by Sternfeld et al. [31] (possible range: 1.0 to 5.0). Domain-specific activity indices were summed to calculate total activity (possible range: 4.0 to 20.0). Total activity was categorized in quartiles.

Medical characteristics including parity (number of previous live births), pre-pregnancy body mass index (BMI) (weight in kg/height in m^2^), family history of type 2 diabetes, personal history of diagnosed GDM, and hypertension in the current pregnancy were abstracted from the medical record. Rate of weight gain up to the time of the GDM screen was calculated by subtracting pre-pregnancy weight from weight at the GDM screen and dividing by the gestational age at the screen.

Dietary intake was assessed among a subsample of participants through a FFQ designed specifically for Hispanics in the Northeastern United States [32]. Dietary variables included total fat, saturated fat, protein, carbohydrate, fiber, total calories, and percent calories from fat, protein, and carbohydrates. Of the 628 completed FFQs, those with more than 12 blank items or with implausible caloric intake (< 600 or > 4500 calories) were excluded, yielding a sample of 566 participants for the diet analysis.

### Data Analysis

Using logistic regression, we calculated odds ratios (ORs) and 95% confidence intervals (CI) for AGT comparing level of cigarette smoking in pre, early, and mid pregnancy to non-smokers in that time period. Age, BMI, and gestational weight gain were *a priori *factors included in multivariable models due to their established associations with AGT [10]. We also independently included each covariate in the model; those factors that changed the association between smoking and AGT by 10% or more (i.e., parity and education) were included in the final multivariable models. The analysis of pre-pregnancy smoking was adjusted for smoking during pregnancy. In order to retain a consistent sample size between age-adjusted and multivariable analyses, women with missing information on any covariate (e.g., age, BMI, weight gain, parity, and education) were retained in the model by assigning their covariate category to 'missing'.

We then examined the association between smoking category and screening glucose from the 1-hr OGTT as a continuous outcome variable. After verifying normality of glucose values, we used multivariable linear regression to generate age-adjusted and adjusted beta coefficients, standard errors, and p values for women in each exposure category (former smokers, > 0-9, 10+ cigarettes per day) as compared to non-smokers.

Finally, we conducted a sub-analysis among the women with dietary data to examine if dietary variables confounded the association between smoking and AGT.

All analyses were conducted using the Statistical Analysis System (SAS Institute, Cary, NC) version 9.1.

## Results

The majority of women were young (72% > 25 years), 55% had not graduated from high school and 60% had an annual household income less than $15,000 (Table 1). The population had a number of risk factors for AGT including overweight or obesity (49%) and family history of diabetes (64%).

**Table 1:** Distribution of sociodemographic, medical, acculturation, and behavioral characteristics according to abnormal glucose tolerance (AGT); Latina Pregnancy Study, 2000-2004

	Total		AGT cases			
		
			Yes		No	
	N	%	N	%	N	%
Total	1006	100.0	119	11.8	887	88.2
Sociodemographic Characteristics						
Age (years)						
16-19	340	33.8	23	19.3	317	35.7
20-24	381	37.9	39	32.8	342	38.6
25-29	177	17.6	26	21.9	151	17.0
30-40	108	10.7	31	26.1	77	8.7
						
Education						
< High School	502	55.2	47	45.2	455	56.5
High/Trade/Technical School	293	32.2	34	32.7	259	32.2
Some College/Graduate	114	12.5	23	22.1	91	11.3
						
Annual household income						
< $15,000	333	59.5	44	57.9	289	59.7
$15,000-30,000	171	30.5	18	23.7	153	31.6
> $30,000	56	10.0	14	18.4	42	8.7
						
Medical Characteristics						
Parity						
0	393	39.2	36	30.5	357	40.3
1-2	491	49.0	491	54.2	427	48.3
3+	119	11.9	18	15.3	101	11.4
Pre-pregnancy BMI (kg/m^2^)						
< 20	128	13.0	10	8.7	118	13.6
20-24.99	371	37.6	26	22.6	345	39.5
25-29.99	245	24.8	32	27.8	213	24.5
30+	242	24.5	47	40.9	195	22.4
Family history of diabetes						
No	339	35.8	25	22.7	314	37.5
Yes	609	64.2	85	77.3	524	62.5
Personal history of GDM						
No	956	95.9	107	91.4	849	96.5
Yes	41	4.1	10	8.6	31	3.5
Weight gain up to glucose screen^1 ^(lbs/week)						
0-< 0.5	253	31.4	29	33.7	224	31.2
0.5-< 1.0	367	45.6	37	43.0	330	45.9
1.0+	185	23.0	20	23.3	165	22.9
Current hypertension						
No	961	95.5	109	91.6	852	96.0
Yes	45	4.5	10	8.4	35	4.0
						
Acculturation Variables						
Language preference						
English Only	655	66.2	69	58.5	586	67.2
Both English and Spanish	141	14.2	22	18.6	119	13.6
Spanish Only	194	19.6	27	22.9	167	19.2
						
Birthplace						
United States	488	53.3	52	49.5	436	53.8
Other	427	46.7	53	50.5	374	46.2
						
Pregnancy Behaviors						
Alcohol Consumption						
No	921	98.4	110	99.1	811	98.3
Yes (1+ drink/week)	15	1.6	1	0.9	14	1.7
Pregnancy physical activity						
1^st ^quartile	227	25.9	25	24.3	202	26.1
2^nd ^quartile	226	25.8	22	21.4	204	26.4
3^rd ^quartile	213	24.3	24	23.3	189	24.5
4th quartile	210	24.0	32	31.0	178	23.0

^1^Rate of weight gain was analyzed as a continuous variable in multivariate models but is presented in categories here.

Note: Sums may not add to 1006 due to missing data for some covariates

A total of 119 (11.8%) participants were classified with AGT. Higher levels of age, education, income, pre-pregnancy BMI, family history of type 2 diabetes, and personal history of GDM were associated with increased risk of AGT (Table 1). In addition, women with AGT were more likely to have hypertension in the current pregnancy. Parity, weight gain up to the time of GDM screen, language preference, birthplace, alcohol consumption and pregnancy physical activity were not associated with AGT.

Approximately one-third of participants (35%) reported smoking cigarettes prior to pregnancy, while 20% of women reported smoking cigarettes during early pregnancy and 17% in mid pregnancy (Table 2). Women who smoked during pregnancy were predominantly light smokers, with 16% smoking > 0-9 cigarettes per day and 4% smoking 10 or more cigarettes per day in early pregnancy. Compared to non-smokers, smokers were more likely to be multiparous, have lower education level and household income, and to drink alcohol during pregnancy (data not shown). Smokers were also more likely to prefer using only English and to be born in the United States.

**Table 2:** Odds ratios of abnormal glucose tolerance (AGT) according to smoking in pre, early, and mid pregnancy; Latina Pregnancy Study, 2000-2004

	Total Population		AGT Cases		Age-adjusted		Age- and weight-gain adjusted		Full Multivariate Model^1^	
					
	No.	%	No.	%	OR	95% CI	OR	95% CI	OR	95% CI
Pre-pregnancy^2^										
Non-smoker	596	65.3	70	11.7	1.00	referent	1.00	referent	1.00	referent
Smoker	316	34.7	35	11.1	1.42	[0.82, 2.46]	1.29	[0.69, 2.41]	1.27	[0.67, 2.38]
> 0-9 cigarettes/day	125	13.7	20	16.0	1.90	[1.02, 3.55]	1.73	[0.84, 3.58]	1.75	[0.83, 3.67]
10+ cigarettes/day	191	20.9	15	7.9	0.95	[0.45, 2.00]	0.89	[0.38, 2.07]	0.86	[0.37, 2.01]
Total	912	100.0	105	11.5						
										
Early Pregnancy										
Non-smoker^3^	524	61.8	62	11.8	1.00	referent	1.00	referent	1.00	referent
Former smoker^4^	153	18.1	24	15.7	1.50	[0.89, 2.52]	1.38	[0.76, 2.51]	1.28	[0.68, 2.41]
Smoker	170	20.1	11	6.5	0.49	[0.25, 0.94]	0.45	[0.20, 1.02]	0.48	[0.21, 1.10]
> 0-9 cigarettes/day	133	15.7	9	6.8	0.59	[0.28, 1.22]	0.49	[0.19, 1.29]	0.49	[0.19, 1.32]
10+ cigarettes/day	37	4.4	2	5.4	0.39	[0.09, 1.67]	0.49	[0.11, 2.18]	0.57	[0.12, 2.66]
Total	847	100.0	97	11.5						
										
Mid Pregnancy										
Non-smoker^3^	387	55.5	43	11.1	1.00	referent	1.00	referent	1.00	referent
Former smoker^5^	193	27.7	29	15.0	1.47	[0.87, 2.48]	1.16	[0.64, 2.10]	1.06	[0.57, 2.00]
Smoker	117	16.8	7	6.0	0.46	[0.20, 1.03]	0.36	[0.12, 1.02]	0.38	[0.13, 1.11]
> 0-9 cigarettes/day	93	13.3	6	6.5	0.62	[0.25, 1.53]	0.35	[0.10, 1.20]	0.36	[0.11, 1.24]
10+ cigarettes/day	24	3.4	1	4.2	0.27	[0.04, 2.08]	0.46	[0.06, 3.79]	0.53	[0.06, 4.67]
Total	697	100.0	79	11.3						

^1^Multivariate model includes age, BMI, weight gain, parity, and education.

^2^Pre pregnancy analysis is also adjusted for early pregnancy smoking.

^3^Current non-smokers who did not previously smoke.

^4^Smoked in pre only; includes non-smokers in early pregnancy with unknown pre-pregnancy smoking status.

^5^Smoked in pre and/or early only; includes non-smokers in mid pregnancy with unknown pre or early smoking status.

After adjusting for age and early pregnancy smoking, women who smoked in pre-pregnancy did not have an increased risk of AGT (OR = 1.42; 95% CI 0.82-2.46) as compared to non-smokers. However, when evaluated according to dose, women who smoked > 0-9 cigarettes per day in pre-pregnancy had an increased risk of AGT (OR = 1.90; 95% 1.02-3.55) compared to pre-pregnancy non-smokers, while those who smoked 10+ cigarettes per day were not at increased risk (OR = 0.95; 95% 0.45-2.00) (Table 2). The increased risk for light smoking during pre-pregnancy was attenuated after adjustment for gestational weight gain (OR = 1.73; 95% CI 0.84-3.58). Similarly, smokers in early (OR = 0.48; 95% CI 0.21, 1.10) and mid pregnancy (OR = 0.38; 95% CI 0.13-1.11) did not have an increased risk of AGT as compared to non-smokers in multivariable models. When we restricted the analysis to participants with complete smoking data in all three time periods (n = 609) findings were virtually unchanged.

We then examined the association between smoking category and continuous glucose levels from the 1-hr OGTT (mean = 101.9 mg/dL) (Table 3). Regardless of dose, pre-pregnancy smoking was not associated with glucose level in age-adjusted and multivariable (β = -2.00, p = 0.36) models. However, women who smoked in early pregnancy (β = -4.60, p = 0.03) and in mid-pregnancy (β = -6.08, p = 0.01) had lower mean glucose levels as compared to non-smokers after adjusting for AGT risk factors.

**Table 3:** Linear regression of one-hour oral glucose tolerance test (1-hr OGTT) plasma values (mg/dL) on smoking status, Latina Pregnancy Study, 2000-2004

	Age-adjusted			Age- and weight-gain adjusted			Multivariate^a^		
			
	Beta	SE	p-value^b^	Beta	SE	p-value^b^	Beta	SE	p-value^b^
Pre-pregnancy									
Non-smoker	referent						referent		
Smoker	-1.37	2.17	*0.53*	-2.86	-2.31	*0.21*	-2.00	2.19	*0.36*
> 0-9 cigarettes/day	0.51	2.58	*0.84*	-2.37	2.77	*0.39*	-0.72	2.62	*0.78*
10+ cigarettes/day	-2.28	2.62	*0.38*	-3.35	2.78	*0.23*	-3.31	2.62	*0.21*
									
Early Pregnancy									
Non-smoker^1^	referent						referent		
Former smoker^2^	-1.00	2.13	*0.64*	-2.73	2.28	*0.23*	-2.46	2.22	*0.27*
Smoker	-5.47	1.99	*< 0.01*	-4.81	2.24	*0.03*	-4.60	2.08	*0.03*
> 0-9 cigarettes/day	-5.61	2.27	*0.01*	-5.54	2.55	*0.03*	-5.16	2.31	*0.03*
10+ cigarettes/day	-6.00	3.92	*0.13*	-5.25	4.35	*0.23*	-5.23	4.19	*0.21*
									
Mid Pregnancy									
Non-smoker^1^	referent						referent		
Former smoker^3^	-1.21	2.06	*0.55*	-2.86	2.23	*0.20*	-2.04	2.06	*0.32*
Smoker	-6.42	2.39	*< 0.01*	-5.99	2.66	*0.02*	-6.08	2.42	*0.01*
> 0-9 cigarettes/day	-6.62	2.73	*0.01*	-7.13	2.96	*0.02*	-6.93	2.73	*0.01*
10+ cigarettes/day	-7.55	4.91	*0.12*	-6.09	6.37	*0.34*	-6.00	5.05	*0.23*

^1^Current non-smokers who did not previously smoke.

^2^Smoked in pre only; includes non-smokers in early pregnancy with unknown pre-pregnancy smoking status.

^3^Smoked in pre and/or early only; includes non-smokers in mid pregnancy with unknown pre or early smoking status.

^a^Adjusted for age, BMI, education, parity, and birthplace. Pre-pregnancy model also adjusted for early pregnancy smoking (yes/no).

^b^The reference group for statistical comparisons was non-smokers who did not previously smoke.

Finally, to determine if the association between smoking and risk of AGT was confounded by dietary intake, we evaluated the association between smoking during pregnancy and AGT in the subset of women with complete dietary intake information (n = 566). Smokers consumed significantly more total fat (p = 0.004) and saturated fat (p = 0.0003) and significantly less fiber (p = 0.005), carbohydrates (p = 0.01), and fewer calories (p = 0.05) than non-smokers. After adjusting for dietary factors, findings were not substantively changed. For example, the multivariable OR for early pregnancy smokers as compared to non-smokers was 0.48 (95% CI 0.18, 1.30).

Women who were not screened for GDM (n = 54) did not differ from those who were screened for GDM in terms of age, parity, education, BMI, family history of diabetes, history of GDM, language preference, or smoking in pre or early pregnancy (all p > 0.20). Similarly, women missing smoking data during any pregnancy time period did not differ from women missing such data during other pregnancy time periods, nor from women with complete information on smoking.

## Discussion

In this prospective cohort study of Hispanic women, results do not suggest an increased risk of AGT related to smoking cigarettes. There was the suggestion that light smoking in pre-pregnancy was associated with an increased risk of AGT compared to non-smokers. Smoking during early and mid pregnancy was not associated with AGT. Cigarette smoking during early and mid pregnancy were associated with lower mean screening glucose values on the 1-hr OGTT glucose tolerance test as compared to non-smokers, independent of weight gain during pregnancy.

Our findings of a suggestion of an increased risk for pre-pregnancy light smoking is consistent with findings among 14,613 participants in the Nurses Health Study [33]. Specifically, Solomon et al. found that, compared to non-smokers, pregravid smokers of 5+ cigarettes per day had an increased risk of GDM (RR = 1.65; 95% CI 1.05-2.58) compared to pregravid non-smokers. It is possible, however, that observed increased risks among pre-pregnancy smokers may be due to the effect of quitting smoking. Quitting or reducing the number of cigarettes smoked during pregnancy may lead to increased gestational weight gain [34], a risk factor for AGT and GDM [35]. Indeed, after controlling for gestational weight gain, our observed increased risk for pre-pregnancy smoking was attenuated.

Similarly, we observed that smoking in early and mid pregnancy were not associated with AGT in age-adjusted or multivariable analyses. Prior epidemiologic studies have been conflicting. In a recent review article, Wendland et al. obtained a crude odds ratio of 1.03 (99% CI 0.85-1.25) and an adjusted odds ratio of 0.95 (95% CI 0.85-1.07) for smoking during pregnancy and risk of GDM [9]. In the only prior study to assess Hispanic women (Brazil), Wendland et al. found that women who smoked during pregnancy had a reduction in risk of GDM (OR = 0.69; 95% CI 0.50-0.96) after adjusting for GDM risk factors [21]. While there were no significant differences in fasting and 1 h plasma glucose means, those for 2 h glucose were significantly lower in smokers (5.58+0.04 mmol/L), p < 0.01) in adjusted analyses. Similarly, we found that smokers in early and mid pregnancy had lower levels of plasma screening glucose values as compared to non-smokers.

There is biologic plausibility for such an association. Nicotine has been shown to block the release of inflammatory cytokines, inhibiting inflammation associated with insulin resistance and thereby diminishing hyperglycemia [9]. Smoking-induced release of catecholamines may also increase the metabolism of soluble nutrients, thereby lowering glucose levels among smokers [36]. Smoking has also been consistently and negatively associated with pre-eclampsia via physiological mechanisms that may be similar [37]. These observations, however, do not exclude the fact that smoking may increase risk of type 2 diabetes in non pregnant women through different metabolic pathways. In addition, the higher doses of smoking during the pre- or non-pregnancy periods [38] may explain, in part, differences in associations between pre-pregnancy vs. pregnancy smoking and risk of AGT. Finally, it is important to note the established adverse effects of smoking on birth outcomes.

Timing of smoking measurement during pregnancy may also explain, in part, differences in study findings. This meta analysis found that studies that assessed smoking earlier in pregnancy [11,13-15,18] showed a tendency to present an increased risk of GDM, while studies that ascertained smoking later [10,16,19] showed odds ratios slightly less than 1. The authors attribute elevated risks observed in early pregnancy to failure to identify smokers who quit or reduced the number of cigarettes smoked, therefore erroneously attributing the association found to smoking rather than to its reduction and subsequent possible increase in weight gain. Smoking assessed later in pregnancy may therefore be a more accurate assessment of true smoking status in pregnancy.

Our study had several limitations. The relatively small number of cases of AGT likely limited our power to detect associations in analyses of AGT as a dichotomous outcome and also precluded evaluating risk of frank GDM. However, this limitation was not faced by the analysis of screening glucose level as a continuous outcome. Like all studies relying on self-reported smoking information, there may be some degree of misclassification in the smoking variable. However, while women may be more susceptible to biased reporting in smoking cessation trials [39], evidence suggests that women are truthful when self-reporting this information in observational studies [40-43]. A study by Christensen et al. [42] also using a structured, interviewer-administered questionnaire found high validity for self-reported smoking when compared to exhaled carbon monoxide levels. Ross et al. [40] compared self-reported cigarette smoking with serum cotinine levels among pregnant women in Minnesota and found that 96% of women who denied smoking reported their status correctly (kappa = 0.86). The validity of self-reported smoking during pregnancy has not been studied in Hispanic women. However, the relatively high smoking rates observed in the current study (35% pre-pregnancy, 20% early pregnancy) suggest that underreporting of smoking may be less of a concern. In addition, by incorporating a category for former smokers in our analysis, we avoid a limitation faced by prior studies that combined ex-smokers with never smokers [9].

Although we had extensive information on established risk factors for AGT, dietary variables were only available for a subset of women who completed the FFQ at the mid pregnancy interview. However, findings in this group were consistent with overall study findings. Similarly, women who were not screened for GDM did not differ from screened women in terms of any demographic, health, or smoking variables. Women who were missing information on smoking did not differ from women who were not missing this information.

Participants in this study were healthy Hispanic women with a singleton pregnancy receiving prenatal care. Because participants were predominantly light smokers, we were not able to evaluate the effect of heavy smoking on AGT. There is also some evidence that nicotine metabolism varies by race [22,23]. Therefore, care should be taken in generalizing to other racial or ethnic groups whose smoking and AGT patterns may differ. Indeed, the cigarette brands, tar content, inhalation behavior, age of first use, and overall patterns of smoking among Hispanics may differ from other populations [24,28], and these characteristics of cigarette use among Hispanics may impact the effect of smoking on AGT.

Finally, BMI, parity, age, and gestational weight gain may be important effect modifiers of the association between smoking and AGT [9]. However, due to our limited sample size we did not have adequate power to assess effect modification by these factors with sufficient precision. Future studies in Puerto Rican women should evaluate the effect of these variables.

## Conclusions

To our knowledge this is the first study to examine the association between smoking and AGT among a Puerto Rican population. This study contributes to the sparse literature on smoking and AGT. We did not observe any increased risk of AGT associated with smoking during pregnancy; findings should be confirmed in other study populations.

## Declaration of Competing interests

The authors declare that they have no competing interests.

## Authors' contributions

AH conceived of the study question, analyzed the data and drafted the manuscript. EB, PP, and EC assisted with data analysis, interpretation, and manuscript editing. RT assisted with data analysis. LC designed the study and assisted with data analysis, interpretation and manuscript editing. All authors read and approved the final manuscript.

## Pre-publication history

The pre-publication history for this paper can be accessed here:

http://www.biomedcentral.com/1471-2393/10/55/prepub
